# New insights into the genomic landscape of meningiomas identified FGFR3 in a subset of patients with favorable prognoses

**DOI:** 10.18632/oncotarget.27178

**Published:** 2019-09-17

**Authors:** Aysha AlSahlawi, Rasha Aljelaify, Amna Magrashi, Mariam AlSaeed, Amal Almutairi, Fatimah Alqubaishi, Abdulellah Alturkistani, Abdullah AlObaid, Mohamed Abouelhoda, Latifa AlMubarak, Nada AlTassan, Malak Abedalthagafi

**Affiliations:** ^1^ Genomics Research Department, Saudi Human Genome Project, King Fahad Medical City and King Abdulaziz City for Science and Technology, Riyadh, Saudi Arabia; ^2^ Montreal Neurological Institute, Montreal, Canada; ^3^ Neurosurgery Department, King Fahad Medical City, Riyadh, Saudi Arabia; ^4^ Saudi Human Genome Program, King Abdulaziz City for Science and Technology (KACST), Riyadh, Saudi Arabia; ^5^ Genetics Department, King Faisal Specialists Hospital and Research Center, Riyadh, Saudi Arabia

**Keywords:** meningioma, FGFR3, NGS, genomics, CNS

## Abstract

**Background:** With a prevalence of 170 000 adults in the US alone, meningiomas are the most common primary intracranial tumors. The management of skull base meningiomas is challenging due to their complexity and proximity to crucial nearby structures. The identification of oncogenic mutations has provided further insights into the tumorigenesis of meningioma and the possibility of targeted therapy.

This study aimed to further investigate the association of mutational profiles with anatomical distribution, histological subtype, WHO grade, and recurrence in patients with meningioma.

**Methods:** Tissue samples were collected from 71 patients diagnosed with meningioma from 2008 to 2016. A total of 51 cases were skull based. Samples were subjected to targeted sequencing using a next generation customized cancer gene panel (*n* = 66 genes analyzed).

**Results:** We detected genomic alterations (GAs) in 68 tumors, averaging 1.56 ± 1.07 genomic alterations (GAs) per sample. *NF2* was the most frequently altered gene (36/71 cases). Interestingly, we identified a number of mutations in non-*NF2* genes, including a hotspot *TERTp* c.−124: G > A mutation that may be related to poor prognosis and *FGFR3* mutations that may represent biomarkers of a favorable prognosis as reported in other cancers.

**Conclusions:** We demonstrate that comprehensive genomic profiling in our population can reveal a potential new prognostic biomarkers of skull base meningioma. These mutations can enhance diagnostic accuracy and clinical decision-making. Among our findings were the identification of a *TERTp* mutation and the first report of *FGFR3* mutations that may represent biomarkers for the identification of skull base meningioma patients with a favorable prognosis.

## INTRODUCTION

With a prevalence of 170 000 adults in the US alone, meningioma is now recognized as the most common primary intracranial tumor [1, 2]. Typically, meningiomas arise from the outermost layer of arachnoid mater cap cells [3]. Although most are benign, approximately 20% show aggressive behavior with 5-year recurrence rates of 50-80% in grade II and III cases [4]. Complete surgical resection is the primary goal during disease management; however, this is only achieved in ~50% of cases due the anatomical complexity and proximity to crucial nearby structures, particularly in tumors occupying the skull base [5, 6]. As approximately 25% of intracranial meningiomas involve the petroclival region, cavernous sinus, temporal fossa, and foramen magnum, complete surgical resection by neurosurgeons is challenging [3]. The identification of predictors of meningioma recurrence is critical to advance our understanding of tumorigenesis and improve the possibility of targeted therapy for these patients [7–15]. This is important since high-grade meningiomas frequently recur and are associated with high rates of morbidity and mortality [16].

Since the introduction of next generation sequencing (NGS), brain tumors are classified based on their molecular parameters as reported in the 2016 World Health Organization (WHO) classification [17]. However, for meningiomas, histological features remain the main differentiating factor, as our understanding of the genomic aberrations that drive these tumors remains incomplete. The 2016 World Health Organization (WHO) classification classes meningiomas into 3 grades and ~15 histopathological subtypes. Tumors are classed as WHO grade I–III based on mitotic activity, neuronal invasion and other aggressive features (e.g. macronuclei, hypercellularity and necrosis). Specific histologies are used to specify tumor grade such as those with clear-cell or chordoid histological morphologies are defined as grade II, whilst papillary meningiomas are classified as grade III. The controversy in these grading systems necessitates the need for more detailed investigations to identify mutational markers, which can be further integrated into combined histological-molecular classifications.

In this regard, Neurofibromin 2 (merlin, NF2) is recognized as the main tumor suppressor gene in meningioma, as it is observed in 40 to 60% of early-stage tumors [18, 19]. *NF2* non-mutated tumors are reported to express other isolated chromosomal alterations and gene mutations at relatively high frequencies [20]. Most commonly, *TNF* receptor-associated factor 7 (*TRAF7*), Smoothened, frizzled family receptor (*SMO*), Krupple-like factor 4 (*KLF4*), and v-akt murine thymoma viral oncogene homolog 1 (*AKT1*) have been identified. More recently, a mutation in Phosphatidylinositol 4,5-bisphosphate 3-kinase catalytic subunit alpha isoform (*PIK3CA*) was detected by targeted sequencing of 150 meningiomas [21]. When investigating the correlation between clinicopathological features and genotype, 80% of *NF2*-mutated meningiomas were found in the calvarium. To date, skull base lesions have not been shown to express other non-*NF2* mutations [22].

In Saudi Arabia, the prevalence of brain tumors is approximately 0.3%; the second highest in the Middle East [23]. In studies assessing the epidemiology of primary brain tumors in Saudi Arabia, grade I meningiomas are the most commonly diagnosed pathological type, with overall recurrence rates of between 10.5 to 22.0% [24]. In this study, we reveal key genetic hotspots that contribute to the tumorigenesis and progression of meningioma in Saudi Arabia with special focus on skull based cases. We identified novel mutations in non-*NF2* skull base tumors that may be related to tumor prognosis including Fibroblast growth receptor-3 (*FGFR3*). Mutations in this gene have not been demonstrated in human CNS tumors, despite its implications in several other forms of neoplasia.

## RESULTS

### Cohort demographics

**Figure 1:**
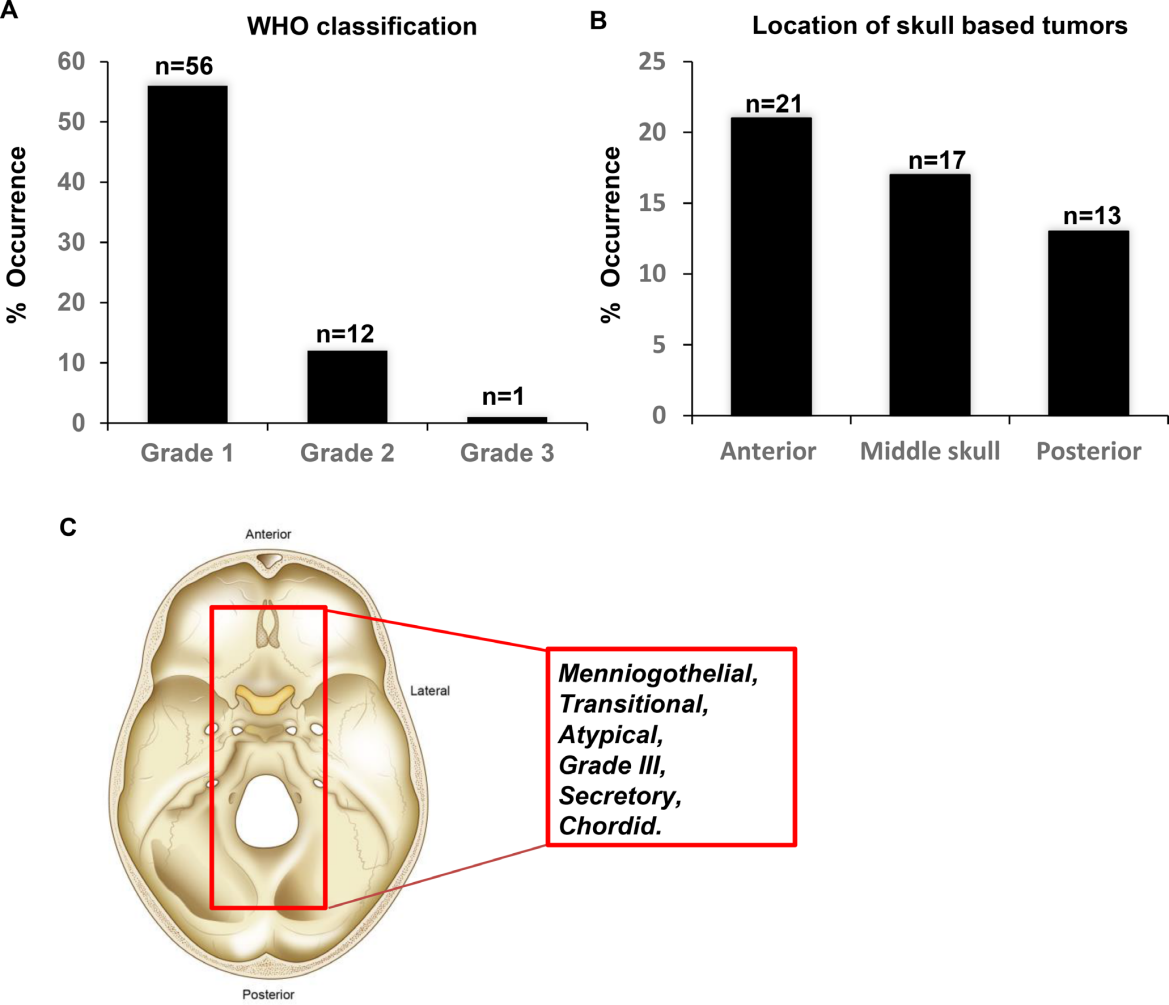
Characteristics of the meningioma’s investigated in the study. (**A**) Tumor grade according to the most recent WHO classification. (**B**) Location of the skull based tumors. (**C**) Schematic demonstrating the histology of the identified tumors from the study cohort.

The study cohort comprised 71 patients with histologically proven meningioma (grades I, II, and III) reviewed by a board-certified neuropathologist (MA). The median patient age was 54.8 years (range 27–96 years). Most of the patients were female (51/71, 71.83%), while 20/71 (28.17%) were male. According to the WHO grade analysis, 57/71 patients had grade I (80.28%), 13/71 (18.30%) had grade II and 1/71 (1.420%) had grade III tumors (Figure 1A). A total of 12/71 patients (17.14%) received radiation therapy. A total of 51/71 of the tumors were found in the skull base (71.83%). Of these, 21 were anterior, 17 were middle and 13 were posterior tumors (Figure 1B). A total of 60/71 (84.51%) were primary tumors, and 10/71 were recurrent tumors (14.08%). According to WHO criteria, 22/71 (31.42%) of the tumors were meningothelial, 6/71 (8.57%) were atypical, 6/71 (8.57%) were transitional, 6 were grade f (8.57%), 3/71 (4.28) were secretory, and 2/71 (2.86%) were chordoid (Figure 1C). The patient characteristics are summarized in Figure 1 and Supplementary Table 1.

### NGS analysis

**Figure 2:**
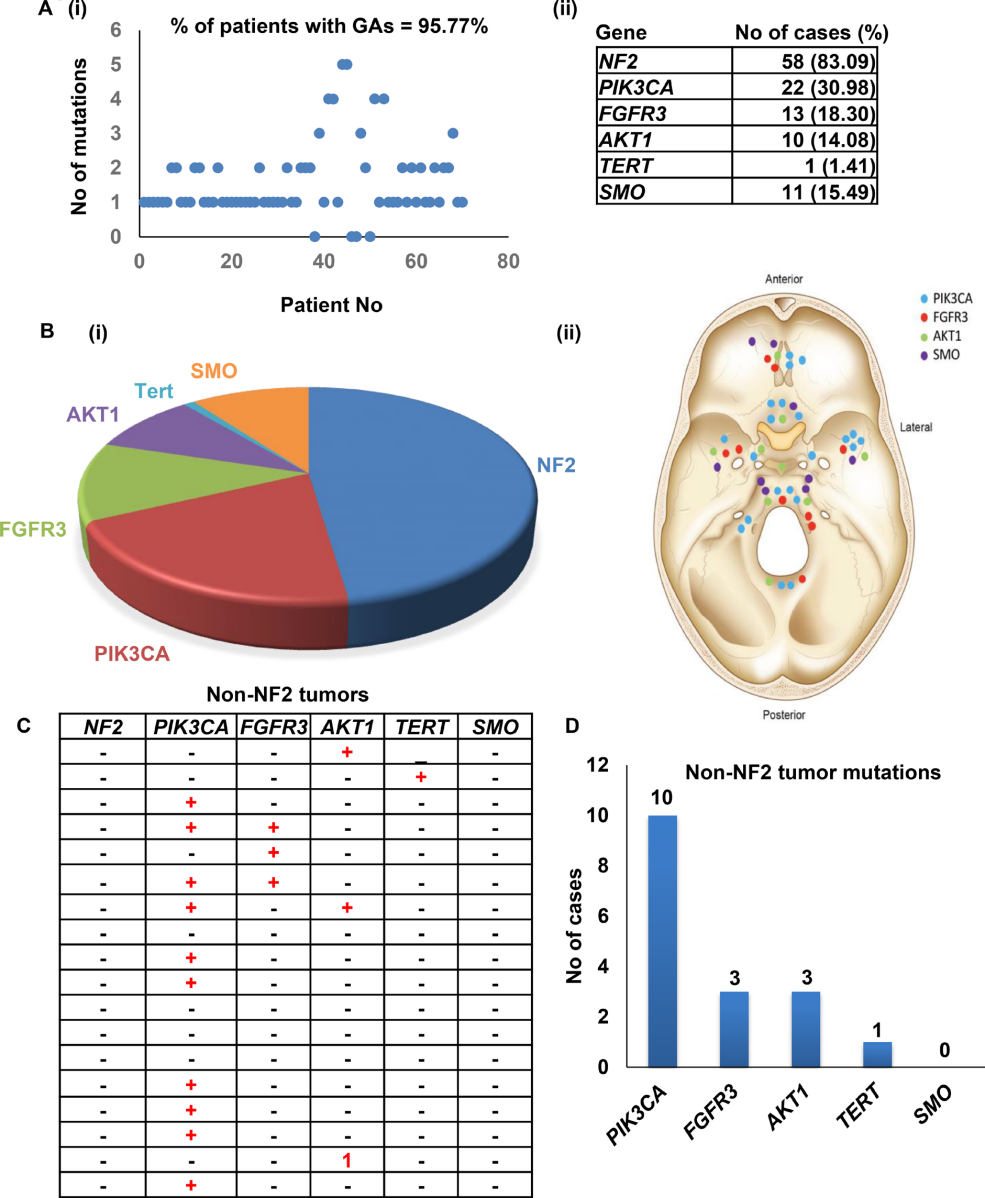
Meningioma mutations identified in the study. (**A**) Frequencies of genomic alterations identified in the study cohort. (**B**) Number of patients harboring the most commonly identified mutations. (**C**–**D**) Mutational frequencies in non-NF2 tumors.

Mutations were detected in 68/71 patients (95.77%) with an average of 1.56 ± 1.07 genomic alterations (GAs) per patient (Figure 2B, Supplementary Table 2). Novel mutations found in three genes *NF2, FGFR3* and *PIK3CA*. For NF2, 35 cases carried the missense mutation c.1060G>A (p.Ala354Thr), However, two of the 35 cases carried known pathogenic stop-gained mutation: c.784C>T (p.Arg262Ter). Another two novel missense mutations: c.925G>A (p.Glu309Lys) and c.1376T>C (p.Leu459Pro) found in two cases. Both mutations were predicted to have a damaging phenotype with PolyPhen-2 HVAR scores equaling 0.909 for c.925G>A (p.Glu309Lys) mutation and 1 for c.1376T>C (p.Leu459Pro) mutation. MutationTaster predictions showed a disease-causing result with a rank score equal to 0.81 for both mutations, while PROVEAN results suggested that both mutations are deleterious with scores equal to -3,32 and -6.32, respectively. For frameshift deletions, one mutation was found in one case.

Two novel missense mutations (c.932T>C (p.Val311Ala) and c.1376G>C (p.Arg459Pro)) in the *FGFR3* gene were found in two cases, and both mutations were predicted to be disease-causing with rank scores equal to 0.81. PolyPhen-2 data suggested that c.1376G>C (p.Arg459Pro) mutation harbored a damaging PolyPhen-2 HVAR score equal to 0.996, with PROVEAN results suggesting a deleterious mutation with a score of -5.80. The other mutation was predicated to be benign and neutral. Both patients were WHO Grade 1, received radiotherapy and showed no tumor recurrence.

For the *PIK3CA* gene, 4 of the cases carried different novel exonic mutations. One case had a missense mutation that was predicted to be a disease-causing by MutationTaster but benign and neutral by PolyPhen-2 and PROVEAN. In addition, six cases were found harbor two reported pathogenic and likely pathogenic mutations; c.3140A>T (p.His1047Leu)/ c.3140A>G (p.His1047Arg) and c.112C>T (p.Arg38Cys). Pathogenic missense mutations in *AKT1(c.49G>A (p.Glu17Lys)) and SMO(c.1234C>T (p.Leu412Phe))* genes were found in 5 and 4 cases, respectively. For the *SMARCB1* gene, one reported synonymous mutation (c.897G>A (p.Ser299=)) that was likely benign was found in 5 cases (Figure 2A and 2B).

NF2 mutations were identified in (58/71) tumors and different patterns of genetic alterations were observed according to the *NF2* status. Tumors with *NF2* mutations harbored 1.83 GAs, and tumors with non-*NF2* mutations harbored 1.24 GAs (Figure 2C). The *NF2*-positive tumors were predominantly of grade I (43/52, 82.69%), which was comparable to tumors lacking *NF2* mutations (14/18, 77.77%). *PI3KCA* was the most frequently mutated gene in the non-*NF2* mutated tumors (Figure 2C and 2D). Importantly, we identified 13 skull base tumors lacking *NF2* mutations.

### Hotspot TERTp mutational analysis

**Figure 3:**
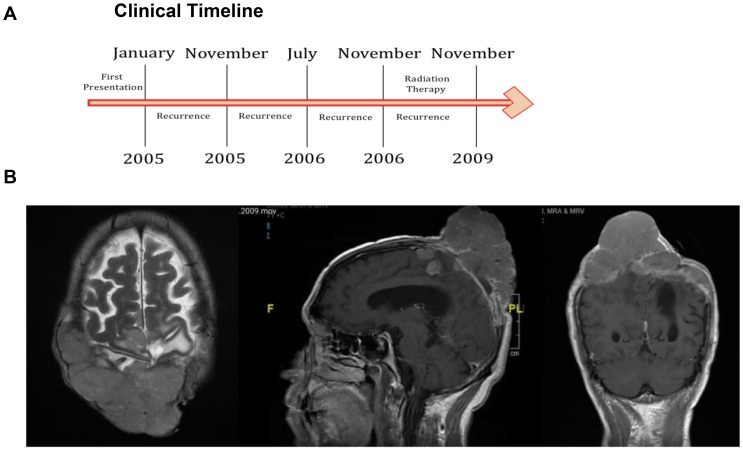
TERTp mutation identified in a single case. (**A**) Clinical timeline of the identified patient. (**B**) Pre-operative MRI of axial, sagittal and coronal views of the patient.

Screening of hotspot *TERTp* mutations, such as c.−146: G > A and c.−124: G > A, identified only a single patient (1/71) affected by the c.−124: G > A mutation. The patient did not possess a c.−146: G > A mutation (clinical timeline in Figure 3A). The patient was a male, aged 68 years, who had recurrent atypical WHO grade II biparietal meningioma. The patient underwent debulking resection. The patient was negative for mutations in *NF2, PIK3CA, FGFR3, AKT1,* and *SMO*. Pre-operative MRI of axial, sagittal and coronal views of the patient demonstrated a biparietal large fungating Meningioma (Figure 3B).

## DISCUSSION

An array of genetic mutations has been proposed as causative for meningioma, including *NF2, MN1, ARID1B, SEMA4D*, and *MUC2. NF2* and *MN1* are classified as driver mutations that play a role in meningioma progression [25]. In this study, 71 tumors from histologically proven meningioma (grades I, II, & III) in the skull base were genetically analyzed. Based on histological analysis, the majority of patients were diagnosed with grade I meningothelial meningioma, and genomic alterations were identified in almost all cases (69/71). Among the meningiomas, the most common mutations were in *NF2* (59/71), *PIK3CA* (22/71), *FGFR3* (13/71), *SMO* (11/71) and *AKT1* (10/71), with *Tertp* (1/71) mutations the least frequent. This was comparable to previous findings in which *NF2* was mutated in ~79% of cases [26]. Of note, we identified 12/71 patients who did not harbor *NF2* alterations [26].

*NF2*-mutated meningiomas show higher chromosomal instability during progression than non-*NF2* mutated meningiomas [27–32]. In this study, tumors with NF2 mutations harbored 1.83 GAs per patient compared to the average of 1.24 GAs in non-*NF2* mutations, consistent with this instability. The *NF2*-positive tumors were predominantly of grade I 43/52 (82.69%), which was comparable to tumors lacking *NF2* mutations (77.77%). Thus, we did not observe an association between the tumor status and *NF2* alterations reported in other cohorts [29]. Tumors in the skull base are traditionally amenable to dissection and debulking with resection. We observed significantly different rates (*p* ≤ 0,05) of recurrence (7/52, 13.41%) in tumors harboring *NF2* mutations compared to tumor harboring non-*NF2* mutations (8/18, 44.44%), suggesting that *NF2* drivers are associated with improved outcomes. This information is useful for appropriate resection and accurate evaluation of meningioma prognosis, highlighting that non-*NF2* tumors may benefit from targeted treatments due to their lower mutation rates but higher likelihood of recurrence. It is therefore important that diagnostic tests are not restricted to frequent *NF2* mutational profiles, and diverse genetic profiling is readily available in clinical settings.

The least frequent mutation was in *TERTp* (1/71 tumors). Telomerases are unique reverse transcriptases that maintain telomere length during cell division [33]. Telomerase activity is robust in embryonic cells but suppressed in mature somatic cells during adulthood. Telomerases are expressed in up to ~90% of solid tumors [34, 35]. Several studies have reported the frequent occurrence of *TERTp* mutations in gliomas that result in altered telomere lengthening and increased tumor survival due to their ability to escape cell senescence [36]. Indeed, the presence of *TERTp* mutations in meningioma is associated with a poorer prognosis and shorter overall survival [1]. We found that TERTp mutations were much less frequent in skull base meningiomas, with grade I tumors showing no incidence (0/56). The *TERTp* mutation was observed in a male patient aged 68 years who had recurrent atypical WHO grade II meningioma and underwent debulking resection. Further genetic profiling of a larger number of higher grade meningiomas that were encountered in this study (only ≥ 12 tumors higher than grade I) will be required to confirm whether *TERTp* indeed directly influences prognosis. If proven to be the case, this subgroup of meningioma patients may require more frequent follow-up and aggressive management to optimize their survival outcomes [1].

We observed alterations in *PI3KCA* (22/71 cases) and *AKT1* (10/71 cases). PI3KCA was the most mutated gene in non-*NF2* tumors. Mutations in these survival genes are typically associated with therapeutic resistance and chemotherapy-induced mutagenesis [37–40]. This highlights the importance of genetic assessment following surgical resection; if *PI3KCA*- and *AKT1*-mutated tumors recur and undergo malignant progression to a higher histological grade, postoperative adjuvant treatment using known and well-characterized inhibitors should be employed, as opposed to radiotherapy or chemotherapy interventions. In this regard, taselisib and fulvestrant (Faslodex) have shown antitumor activity and may be recommended [41–45].

With regard to the potential of our datasets to guide precision therapy, smoothened (encoded by the *SMO* gene) is a Frizzled G protein-coupled receptor and a critically important component of the hedgehog signaling pathway [46]. *SMO* mutations were identified in 11/71 (15.74%) cases in our cohort, leading to a characterized predisposition and vulnerability to meningioma formation [47, 48]. *SMO* is a molecular target of vismodegib, the first small-molecule hedgehog inhibitor to be approved by the FDA [49] that has shown promise in the treatment of basal cell carcinomas [50]. Of note, vismodegib has been included in current clinical trials on progressive meningioma (NCT02523014) in patients harboring SMO mutations. In lung cancer studies, *SMO* amplifications and subsequent activation of the hedgehog pathway confer resistance to anti-EGFR drugs [46]. Phase II trials of erlotinib or gefitinib, two targeted EGFR inhibitors, demonstrated limited efficacy in progressive meningioma patients, thought to be due to the expression of EGFR in the trial participants [51]. We anticipate that the subset of SMO mutation-harboring tumors we identified will show a response to vismodegib therapy, but the efficacy of anti-EGFR therapeutics is unlikely due to drug resistance. Genetically profiling erlotinib- and gefitinib-resistant meningioma would further support these findings.

An interesting occurrence was the identification of single *FGFR3* mutations in three patients. Two novel missense mutations in the *FGFR3* gene were found in two cases, and PolyPhen-2 data suggested that one mutation harbored a damaging PolyPhen-2 score (HVAR score equal to 0.996). Previous studies have highlighted *FGFR3* mutations in an array of malignancies, including breast cancer, bladder cancer, prostate cancer, and squamous non-small cell lung carcinoma (sqNSCLC) [52–57]. Interesting *FGFR3* mutations are typically associated with low-grade cancers and favorable prognoses [55, 56, 58], and patients harboring these mutations had WHO grade I tumors, with no recurrence in our cohort. Clinical trials for sqNSCLC with the selective FGFR1-4 inhibitor erdafitinib are currently ongoing (NCT03827850), and a favorable therapeutic outcome is predicted in FGFR3-mutated meningiomas (Supplementary Figure 1). Thus, *FGFR3* mutations may be a prognostic biomarker for a favorable outcome in meningioma patients, who may further respond to erdafitinib treatment.

## MATERIALS AND METHODS

### Patients and samples

Tissue samples were collected from 71 patients diagnosed with meningioma according to clinical and pathological criteria from 2008 to 2016. Tissues were treated and archived as formalin-fixed paraffin-embedded (FFPE) samples in the Pathology Department, KFMC, Riyadh, KSA. This study was performed with IRB#16-310 following the relevant ethical guidelines and regulations from the King Fahad Medical City, Riyadh, KSA.

### DNA extraction and quantification

FFPE blocks were retrieved from the Pathology Department, KFMC, Riyadh, KSA. Genomic DNA was extracted from FFPE samples using GeneRead DNA FFPE Kits (Qiagen, USA) according to the manufacturer’s protocol. DNA samples were quantified by Qubit^®^ 3.0 Fluorometer (Thermo Fisher Scientific, USA) using the Qubit dsDNA HS Assay Kit (Thermo Fisher Scientific, USA).

### TERT promoter (TERTp) mutational screening

All 71 samples were screened for the presence of hotspot *TERTp* mutations (c.−146: G > A and c.−124: G > A). PCR was performed using the primer pairs Fw: 5′-CAGCGCTGCCTGAAACTC-3′ and Rev: 5′-GTCCTGCCCCTTCACCTT-3′ [7]. HotStarTaq DNA Polymerase PCR kits were used for PCR (Qiagen, USA) following a touchdown PCR program with annealing temperatures of 64 to 55° C, resulting in PCR products of ~235 bp. Products were purified using the Agencourt AMPure PCR purification system (Agencourt Bioscience, USA) and sequenced using the BigDye Terminator Cycle Sequencing kit (PE Applied Biosystems, USA). Runs were performed on ABI 3730xl capillary sequencers as previously described [59].

### Targeted sequencing

All samples underwent targeted sequencing using a customized panel designed by Thermo Fisher Scientific that has been verified for both sensitivity and specificity. The panel includes the following 66 known cancer genes: *HRAS STK11, TERT, FGFR3, CARD11, GNA11, JAK2, TP53, VHL, MYCN, JAK3, CDKN2A, SMARCB1, KRAS, FLT3, ALK, NF1, NF2, SRC, MLH1, ERBB2, FGFR1, DDX3X, CTNNB1, CIC, RET, MPL, MSH2, MSH6, SMAD4, RB1, CYLD, EGFR, PDGFRA, KIT, KDR, GNAS, BCL2, MEN1, CDH1, BCL1, ATRX, GNAQ, PTEN, IDH2, CDK6, PTCH1, AKT1, ATM, APC, PTPN11, NRAS, MET, HNF1A, FGFR2, MYC, SMO, ABL1, NOTCH1, BRAF, FBXW7, NPM1, PIK3CA, BCL6, IDH1,* and *ERBB4.*

Libraries were manually prepared using the Ion AmpliSeq™ Library Kit 2.0 (Thermo Fisher Scientific, USA). Templates were prepared using the KingFisher System (Thermo Fisher Scientific, USA) and Ion AmpliSeq™ Kit for Chef DL8 (Thermo Fisher Scientific, USA) and Ion 530 Chip Kits (Thermo Fisher Scientific, USA). Templates were sequenced using the Ion S5XL system and Ion S5 sequencing solution (Thermo Fisher Scientific, USA). All sequencing experiments were performed in the Saudi Human Genome Project Laboratory, KFMC, Riyadh, KSA.

### Sequence analysis and bioinformatics

Sanger sequencing for *TERTp* mutations was performed using SeqMan NGen® (version 5.07 DNASTAR, USA). The NGS data pipeline was implemented by the bioinformatics teams of the Saudi Human Genome Projects, KACST and KFMC, Riyadh, KSA. Briefly, the quality of the NGS reads were measured, and low-quality reads were excluded. Reads were aligned to the reference genome using Torrent Aligner Software (Torrent Mapping Alignment Programs (TMAP)) and the human genome reference (GRCh37/hg19). Variants were detected for each sequenced sample using the Torrent Variant Caller (TVC) program. Resultant files were saved in the variant call format (VCF). The VCF file was used for annotation against dbSNP, the 1000 Genomes Project, the Human Gene Mutation Database, and the Catalog of Somatic Mutations in Cancer. A local genome database (SGP777) that includes specific Arab variants was compared.

Previously identified exonic mutations with pathogenic effects were reported, whilst mutations with benign effects were excluded. Novel exonic mutations were assessed for the prediction of damage effects using the *in silico* prediction tools MutationTaster (http://www.mutationtaster.org), PolyPhen-2 (http://genetics.bwh.harvard.edu/pph2/index.shtml), and PROVEAN (http://provean.jcvi.org/index.php).

### Statistical analysis

*P*-values < 0.05 were considered statistically significant. Secure electronic databases were created for data storage and analysis. Data were entered and analyzed using SPSS version 23.

## CONCLUSIONS

While our cohort was focused on skull base meningioma’s, investigation of sequencing data in our cohort has identified a number of non-*NF2* mutations in skull base meningioma’s, This highlights a range of mutations outside the known cancer driver *NF2* that may be linked to meningioma prognosis. Taken together, these findings highlight how genetic profiling can guide optimal treatment strategies, prognostic prediction, and patient management for skull based meningioma. Further studies in a range of meningioma grades are now required to confirm the prognostic significance of the identified mutants.

## SUPPLEMENTARY MATERIALS




