# Supplementary material for: New insights into the genomic landscape of meningiomas identified FGFR3 in a subset of patients with favorable prognoses
Source: Oncotarget. 2019 Sep 17;10(53):5549–59. doi: 10.18632/oncotarget.27178 (PMC6756861; doi:10.18632/oncotarget.27178)
Supplement: Supplementary file 1 [file oncotarget-10-5549-s001.pdf]

New insights into the genomic landscape of meningiomas identified FGFR3 in a subset of patients with favorable prognoses

SUPPLEMENTARY MATERIALS

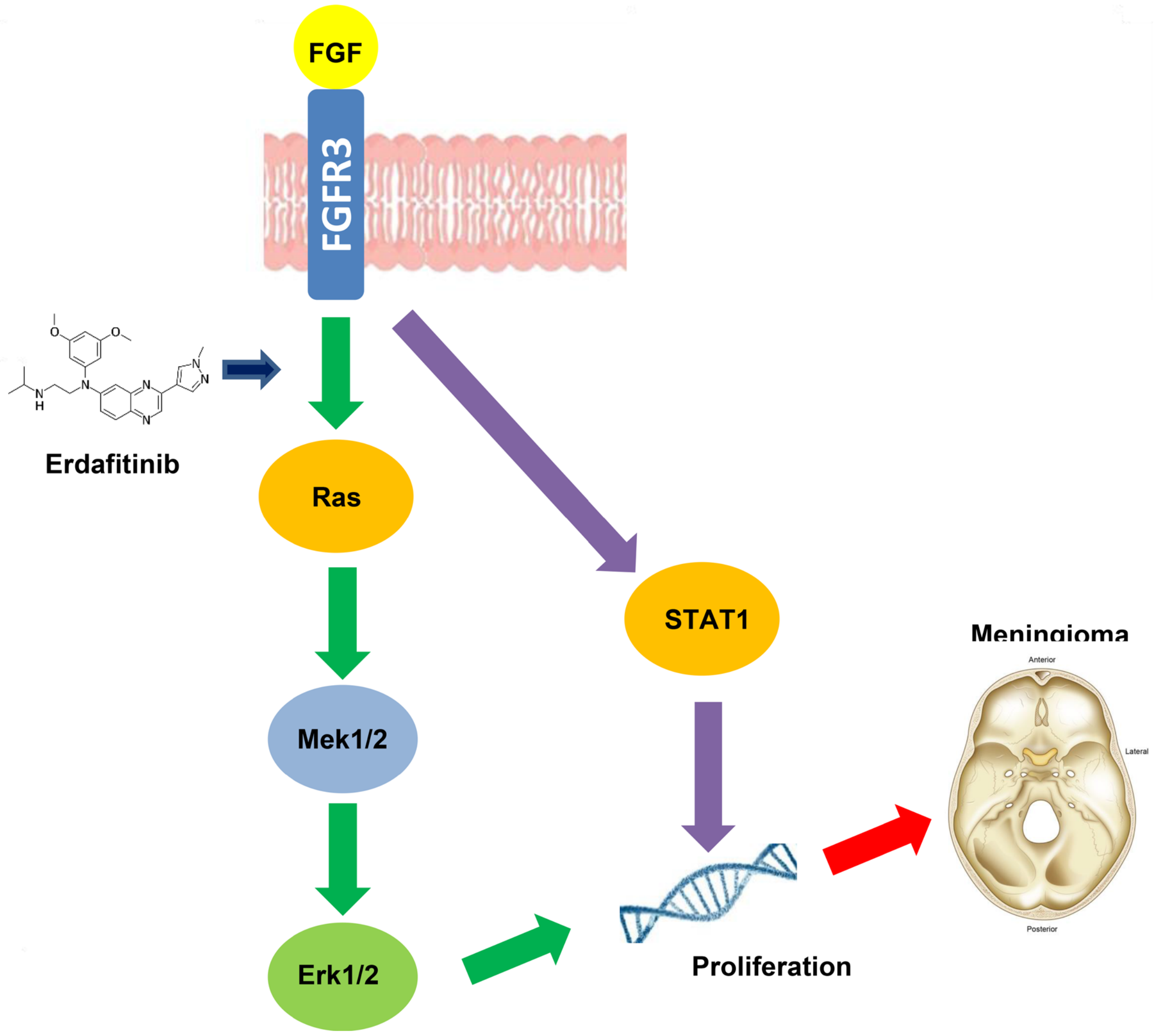

Supplementary Figure 1. Network of FGFR3 kinase reactions which transmit pro-proliferative signals identified in meningioma tumors in the study.

Supplementary Table 1: Demographic characteristics of 71 patients with meningioma's.

**Age, y, mean ± SD/median/range**	54.8 years (range 27–96 years).
**Sex**	
**Female**	51 (71.83%)
**Male**	20 (26.17%)
**Localization**	
**Anterior**	21
**Middle**	17
**Posterior**	13
**Primary tumors**	60 (84.51%)
**Recurrent tumors**	10 (14.08%)
**Grade**	
**1**	57 (80.28 %)
**2**	13 (18.30%)
**3**	1 (1.40%)
**Postoperative irradiation**	12 (16.90%)

Supplementary Table 2: Patient mutational data. See Supplementary_Table_2
